# Kinetic Model of Fluorescein Release through Bioprinted Polylactic Acid Membrane

**DOI:** 10.3390/biomimetics9060342

**Published:** 2024-06-05

**Authors:** Antonio de Nigris, Antonio Minó, Giuseppe Cinelli, Matilde Colella, Francesco Lopez, Luigi Ambrosone

**Affiliations:** 1 Department of Medicine and Health Sciences “V. Tiberio”, University of Molise, Via F. De Sanctis, 86100 Campobasso, Italy; a.denigris1@studenti.unimol.it; 2 Department of Biosciences and Territory (DiBT), University of Molise, Contrada Lappone, Isernia, 86090 Pesche, Italy; antonio.mino@unimol.it; 3 Department of Agriculture, Environment and Food (DiAAA), University of Molise, 86100 Campobasso, Italy; giuseppe.cinelli@gmail.com (G.C.); lopez@unimol.it (F.L.); 4 Department of Biosciences, Biotechnology and Environment (DBBA), University of Bari “Aldo Moro”, Via Orabona 4, 70125 Bari, Italy; matilde.colella@uniba.it

**Keywords:** PLA membrane, diffusion, bioprinter, osmotic swelling, compressed exponential

## Abstract

Polylactic acid (PLA)-based cylindrical membranes for the controlled release of fluorescein sodium salt (FS) were prepared by bioprinting on systems with an initial FS concentration of 0.003763 gdm^−3^ and 37.63 gdm^−3^, and the drug release process was monitored in a bath at 37 °C. Photographs, acquired at regular intervals during the process, revealed marked osmotic swelling of the polymer. Osmotic swelling consists in the enlargement of the polymer structure and due to the influx of water molecules across the membrane. The cylindrical PLA membrane starts to significantly swell once a certain threshold range is crossed. Important amounts of FS can dissolve under these radically changed circumstances, and the dissolved FS molecules are mobile enough to diffuse out of the cylinder, thus allowing drug release. As a matter of fact, in this investigation, we ascertained that polymer swelling promotes the mass transport phenomenon by altering the conditions for drug dissolution and diffusion, hence facilitating FS release after a specific lag time. Furthermore, in order to compare the release kinetics, the half-release time, $t_{0.5}$, was taken into consideration. The data of this study evidence that, while increasing the initial concentration of FS by three orders of magnitude, the time parameter, $t_{0.5}$, is only reduced by 5/6. In addition, the yield of the release process is drastically reduced due to the strong aggregation ability of the dye. Finally, it is demonstrated that a compressed exponential kinetic model fits the experimental data well despite the varying physical conditions.

## 1. Introduction

Polylactic acid (PLA) is a thermoplastic polyester derived from renewable resources such as corn starch or sugarcane and can be synthesized through the polymerization of lactic acid [1]. The key advantages of PLA for medical applications are its biodegradability and biocompatibility [2], making it suitable for use in biomedical applications [3]. When processed with a bioprinter, PLA-based constructs can closely resemble the extracellular matrix found in natural tissues, facilitating cellular adhesion, proliferation, and tissue integration [4,5]. This biomimetic approach enhances the compatibility of PLA constructs with the biological environment [6]. In addition, many biological structures exhibit unique functionalities that have evolved over millions of years to perform specific tasks efficiently. By leveraging biomimetic design principles, PLA-based structures fabricated with a bioprinter can incorporate similar functionalities [7]. The use of nanosystems in drug delivery has already had a wide influence in a diverse number of scientific fields [8,9]. The polymer also can encapsulate and release organic compounds; in this specific case, PLA is extensively used to develop systems for drug delivery. Indeed, microparticles and nanoparticles of PLA protect drugs from degradation, controlling their release kinetics and improving their bioavailability [3,10,11]. Because of these properties, PLA sutures are widely used in surgery due to their excellent tensile strength [12].

PLA degrades in water over a period of several months to a few years [13]. Notwithstanding the relatively long degradation time, there are still several medical advantages in using PLA as a drug reservoir [14,15]. Indeed, it can be engineered to degrade over a controlled period, allowing for sustained drug release [16,17]. This can be advantageous for medications that require long-term dosing or that need to be released gradually to maintain therapeutic levels in the body [14]. The PLA decomposition typically occurs through the hydrolysis reaction, wherein the ester bonds in the PLA chain are broken down according to the overall reaction PLA + H_2_O → Lactic Acid. During this process, water molecules diffuse into the polymer and disrupt the intermolecular forces holding the polymer chains together. The penetration of the water molecules into the polymer matrix causes the polymer chains to expand with facilitating the release of the drug present in the polymer [18].

Fluorescein dye is the most used fluorescent probe. Fluorescence occurs when atoms in a substance are excited and then almost immediately re-emit electromagnetic radiation, usually visible light. The fluorescence intensity of a fluorescent compound is proportional to its concentration. Accordingly, fluorescence spectroscopy can be analytically used to measure the unknown amount of a material [19,20]. Fluorescein, chemically known as C_20_H_10_Na_2_O_5_, belongs to the xanthene class [21]. It has an excitation peak at 498 nm and an emission peak at 517 nm [22]. Its distinctive greenish-yellow fluorescence under ultraviolet light renders it easily detectable in medical settings. Due to its intrinsic fluorescent properties and selective binding characteristics, FS has revolutionized diagnostic and therapeutic approaches in various medical disciplines [23,24]. Indeed, it takes an indispensable role in facilitating accurate disease diagnosis, guiding surgical interventions, and monitoring treatment efficacy in diverse medical fields, ranging from ophthalmology [25,26] to cardiology [27]. Fluorescein is used in the diagnosis of corneal perforation [28]. Oral or intravenous fluorescein is used in fluorescein angiography to detect diabetic retinopathy and choroidal neovascular membranes [29]. To manufacture medical devices from PLA by bioprinting, the polymer is first melted at temperatures higher than 200 °C and then extruded under high pressure. However, it was established that PLA is a thermoplastic polymer susceptible to rapid degradation during melt processing at high temperature. Accordingly, reprocessing may compromise the performance of polymer and particularly its permeation properties toward drugs [30,31].

When PLA is exposed to high temperatures, it undergoes several structural and chemical changes that affect its mechanical and thermal properties, including the transition from rigid to brittle, decrease in its transparency, and reduction in mechanical properties due to chain breakage. In the presence of oxygen, it can partially oxidize, misaligning the polymer chains. These structural changes in the polymer can alter its permeability toward drugs [32]. The purpose of this study was to monitor the release properties of a PLA membrane fabricated with a bioprinter and to evaluate its permeation ability toward a known dye/drug, such as fluorescein sodium salt (FS) [26,33,34]. The rate of FS release from the membrane was monitored for more than 12 weeks through spectrophotometric and spectrofluorimetric measurements. Regular time interval photographs detected osmotic swelling in the release bath. Finally, in this investigation, an analytical model, implying a predictive determination of the amount of dye released, is proposed.

## 2. Materials and Methods

### 2.1. Materials

Fluorescein sodium salt was purchased from Sigma Aldrich and used without further purification. PLA was a commercial product (white PLA 3D printer filament, Sunlu Industrial Co. Zhuhai, Guangdong, China). Water used for preparing solutions was ultra pure water (UPW) produced by the Milli-Q system. Measurements taken at the beginning and end of the experiments gave pH = 7.2.

### 2.2. Spectrofluorimetric and Spectrophotometric Measurements

Solutions containing low concentrations of FS ($C_{FS}\leq 0.06$ mgdm^−3^) were analyzed by spectrofluorimetry and, to avoid instrument saturation, solutions with higher concentrations ($0.06<C_{FS}\leq 14$ mgdm^−3^) were investigated by UV–Visible spectrophotometry.

The fluorescence measurements were carried out with a Varian fluorimeter. The width of the excitation and emission slits was 5 mm and the bandwidth of the monochromator was 20 nm. The excitation and emissions wavelengths utilized for this study were 490 and 512 nm, respectively.

The absorption measurements were carried out with a Cary 100-Varian UV–Vis spectrophotometer equipped with thermostatted cells. Aqueous samples were placed in rectangular quartz cells of 1 cm path length, and absorption spectra were recorded in the 200–800 nm wavelength region.

The limit of detection (LOD) and limit of quantification (LOQ) were determined according to ICH Guideline [35]. Particularly, spectrophotometric measurements LOD = 0.071 μgdm^−3^ and LOQ = 0.215 μgdm^−3^, in the concentration range from 50 μgdm^−3^ to 15 mgdm^−3^, were calculated. However, fluorimetric measurements exhibited LOD = 0.23 μgdm^−3^ and LOQ = 0.70 μgdm^−3^ ranging from 0 to 50 μgdm^−3^.

### 2.3. Manufacturing of PLA Membrane

A hollow cylinder with internal diameter $d_{i}=0.01$ m, height L=0.01 m, and thickness $h=410\times 10^{-6}$ m was drawn with Autodesk Inventor Pro software and then manufactured by a BIOX Bioprinter (CELLINK-Twin Helix). In particular, 1.50 g of PLA pellets was loaded into a metal cartridge, melted at 210 °C, and extruded through a 410 μm needle at a speed of 5.00 mms^−1^ with the pressure held at 150 kPa. The upper base (cylinder lid) was separately bioprinted and used to close the cylinder after it was filled with FS solution. The printing bed was maintained at room temperature.

The cylinder was not manufactured by molding but rather by direct layer-upon-layer deposition. The bioprinter takes approximately 5 s to lay down a single layer (circumference). Since 24 layers are required for the full height of the cylinder, it follows that 288 s are necessary to fabricate the cylinder. Furthermore, about 60 s are needed to fabricate the cylinder lid.

### 2.4. Calibration Curves

The conversion of optical intensity measurements to dye concentration in the release bath was performed through appropriate calibration curves.

**Figure 1:**
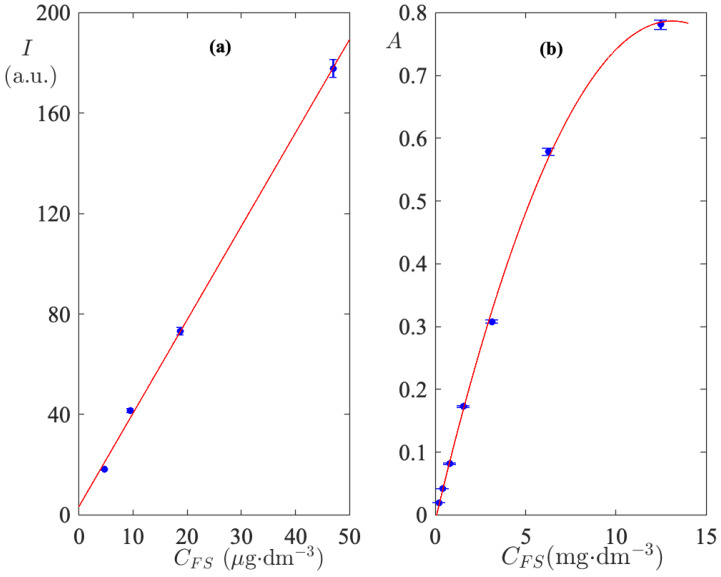
Calibration curves for determining $C_{FS}$ in the release bath from spectroscopic measurements. (**a**) Fluorescence emission intensity employed in the very low concentration range exhibits a linear trend. (**b**) Absorption intensity employed in the range of high concentrations. Nonlinearity is due to strong interactions between dye molecules. Error bars indicate the standard deviation of triplicate measurements.

A number of aqueous FS solutions were prepared and their fluorescence emission spectra were monitored and acquired. The intensity of the emission peak at 512 nm is calculated and plotted as a function of $C_{FS}$ in Figure 1a. As shown in Figure 1a, the fluorescence emission intensity measurements, at 512 nm, for $C_{FS}<0.06$ gdm^−3^ exhibit a linear trend with a correlation coefficient $R^{2}=0.99$. The coefficients of the linear regression were used to extract $C_{FS}$ values from measurements of fluorescence intensity carried out in the release bath. Aqueous solutions of $C_{FS}$ prepared at concentrations higher than 0.06 gdm^−3^ were monitored by UV–Visible absorption spectra. The experimental absorbances of the peak at 489 nm are displayed in Figure 1b. It is immediately clear that at higher concentrations the linearity of the calibration curve is lost. This effect is due to the dye–dye association equilibria which take place in aqueous solutions and are explained elsewhere [26,36,37]. Each point used to construct the calibration curves is the average of triplicate measurements. Their standard deviation is reported as an error bar in the calibration plots in Figure 1a,b. The regression parabola provides coefficients that can convert absorption intensities to concentrations with root mean square error, RMSE=0.01. This value is within the standard deviation of the individual measurements.

Although the initial FS concentrations in the cylinder were very high, the very low permeability of the polymer makes the FS concentration in the release bath suitable to be measured with the calibration curves of Figure 1.

### 2.5. Kinetics Measurements

After filling with the FS solution, the cylinder was closed with the lid and sealed with silicone. Silicone was also chosen to make the diffusion of solvent and solute through the top base of the cylinder impervious. The cylinder loaded with solution was, then, attached by silicone to the bottom of a 0.1 dm^−3^ beaker. After the silicone dried, 0.015 dm^−3^ of UPW was poured into the beaker, which completely covered the cylinder loaded with the dye/drug. The filled cylinder was, then, placed in a shaking water bath oscillating at 30 rpm and operating at 37 °C. At regular time intervals, 0.001 dm^3^ of solution samples was withdrawn from the beaker and fluorimetric or spectrofluorimetric spectra were acquired. In order to avoid changes in the release volume, after analysis the sample was re-introduced into the cylinder. Measurements were performed for initial dye concentrations of 0.03763 and 37.63 gdm^−3^. The initial concentration was increased 1000-fold in order to be able to assess any changes in the permeability of the polymer toward the dye. Indeed, it is well known that the permeability of PLA toward FS is very low; however, the response of the system to a high concentration gradient is unknown.

The monitoring of FS concentration in the release bath was carried out for more than 12 weeks. The poor sensitivity of the spectrophotometer in detecting very low concentrations of dye made it necessary to use spectrofluorimetric analysis to detect $C_{FS}<0.06$ gdm^−3^ and spectrophotometric analysis to determine $C_{FS}>0.06$ gdm^−3^.

## 3. Results and Discussion

### 3.1. Osmotic Swelling

**Figure 2:**
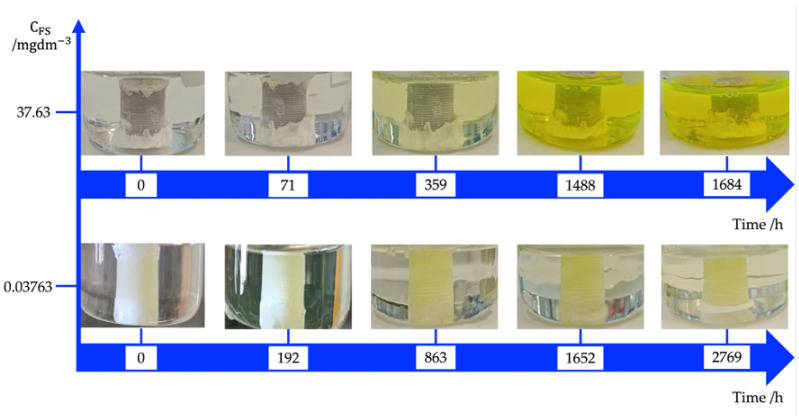
Time evolution of hollow cylinders loaded with FS solution and immersed in release bath at 37 °C. Photographs taken at different time intervals highlights how osmotic swelling is dependent on initial dye concentration.

The photos in Figure 2 depict the time evolution of the solution enclosed in the cylinder. The presence of the dye in the bath outside the cylinder, regardless of the initial concentration of the dye, implies that the PLA membrane is not FS-impermeable.

The hollow cylinder behaves as a membrane that is not selective to either solvent or solute (open membrane). As a result, both water and FS molecules penetrate the membrane. FS molecules are solubilized in the polymer matrix by incorporation into empty spaces, so the higher the level of voids in the membrane, the more dye can be solubilized [38]. An increase in solubilization changes the chemical potential of FS molecules in membranes, alters diffusion through the membrane [17]. Indeed, the driving force for transporting the FS molecules through the membrane is the difference of chemical potential between the starting point and its destination.

**Figure 3:**
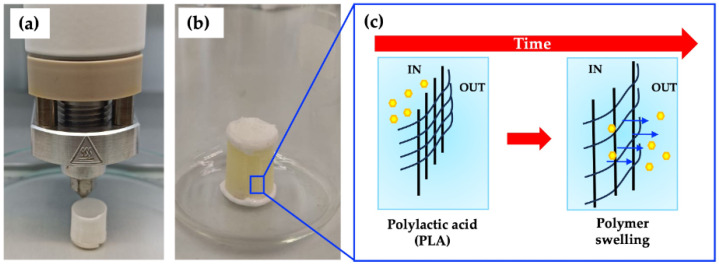
(**a**) Production of hollow cylinder with bioprinter. (**b**) Hollow cylinder loaded with FS solution and immersed in release bath at 37 °C. (**c**) Schematic representation of osmotic swelling of PLA membrane.

As one can see in Figure 2, the residence times are much longer for FS molecules than water. This means that, for short times, the water volume entering in the membrane, is not compensated by the solution leaving, therefore the membrane swells by osmotic effect [39]. As shown in Figure 3a–c, the osmotic effect can alter the stiffness, strength, and toughness of the polymer. The degree of swelling and the resulting changes in properties depend on the interactions between the polymer chains and the solvent molecules. This effect can influence the chemical reactivity of the polymer matrix by altering the accessibility of functional groups or reactive sites within the material. This can impact processes such as degradation and chemical modification. Furthermore, the osmotic effect can be utilized to control the release of drugs from polymeric matrices in drug delivery systems. Indeed, by modulating the swelling behavior of the polymer, the rate and extent of release can be tailored to specific requirements [40].

### 3.2. Kinetic Model

It is assumed that the agitation level of the external bath is high enough to ensure a uniform concentration throughout the release bath, i.e., there are no concentration gradients in the bath. On the other hand, the agitation is not so violent as to reduce to zero the resistance to mass transfer at the membrane surface. Under these hypotheses, the speed with which FS molecules cross the separation surface between the membrane and the release bath would take the form

(1) $$V\frac{dC_{FS}}{dt}=\alpha \int_{B}J_{FS}\left(r,t\right)\hat{n}dS$$

where $C_{FS}$ is the FS concentration in the release bath, *V* is the bath volume, α is the fractional void volume of the solid membrane, and $J_{FS}\left(r,t\right)$ is the FS flow at position r that passes the boundary B at time *t*. The integration is to be taken over the separation surface *S*, and $\hat{n}$ is the unit outward normal to B. As discussed above, relatively large quantities of water enter the membrane due to the osmotic effect (see Figure 1), and this triggers a process of destabilization of the polymer structure.

On the other hand, the ester groups along the polymer chain undergo

(2) $$\text{---}R_{1}COOR_{2}\text{---}+H_{2}O⟶\text{---}\;R_{1}COOH+\text{---}R_{2}OH$$

where R_1_, R_2_ represent chemical groups in the PLA chain and R_1_COOR_2_ highlights the ester group. In addition, PLA is a biodegradable polymer, so, over long times, its mass gradually decreases during immersion in aqueous media.

As is clearly evident from the reaction scheme (Equation (2)), the hydrolysis of PLA produces lactic acid which serves as a catalyst for the hydrolysis reaction itself. The phenomenon, known as self-catalysis, accelerates the process of deterioration of the polymer structure. Water molecules initiate the deconstruction of PLA by generating different adsorption sites for FS molecules. Accordingly, the release of dye through the PLA membrane is not a single process but the result of diffusion, osmosis, adsorption, and chemical reactions.

Some of us (LA) showed that complex systems composed of many chemical species of similar nature with different lifetimes are well described by a continuous distribution of activation energies [41]. On the other hand, a random tridimensional polymer with multiple and chemically variable reactive sites may be considered an example of a complex system; therefore, we consider FS release as an order-one kinetics with time-varying lifetime τ(t), i.e.,

(3) $$\alpha \int_{B}J_{FS}\left(r,t\right)\hat{n}dS=\frac{m_{FS}^{\infty }-m_{FS}}{\tau (t)}$$

where $m_{FS}$ is the mass of FS in a membrane at time *t*, $m_{FS}^{\infty }$ the concentration after infinity time, and τ(t) the timescale of membrane residence.

Then, the substitution of Equation (3) into (1) yields

(4) $$\frac{dm_{FS}}{dt}=\phi \frac{m_{FS}^{\infty }-m_{FS}\left(t\right)}{\tau (t)}=\frac{m_{FS}^{\infty }-m_{FS}\left(t\right)}{\tau_{exp}\left(t\right)}$$

where it is placed

(5) $$\phi =\frac{V_{m}}{V},\;\tau_{exp}\left(t\right)=\frac{\tau (t)}{\phi }$$

$V_{m}$ being the membrane volume and $\tau_{exp}$ the parameter directly accessible to experiments. Equation (5) states that the membrane lifetime is smaller the smaller the membrane volume.

Introducing the dimensionless quantity

(6) $$\chi =\frac{m_{FS}}{m_{FS}^{\infty }}$$

the formal solution of Equation (4), under the initial condition χ(0)=0, leads to

(7) $$\chi \left(t\right)=1-exp\left(-\int_{0}^{t}\frac{dt^{\prime }}{\tau_{exp}\left(t^{\prime }\right)}\;dt^{\prime }\right)$$

In order to obtain an analytical equation for monitoring FS release, it is necessary to know the function $\tau_{exp}\left(t\right)$. For this purpose, we note that PLA is an erodible polymer, so the parameter ϕ can also change over time *t*. We assume that the complexity of the system can be well described by a power law

(8) $$\tau_{exp}\left(t\right)=Bt^{1-\sigma }$$

This immediately leads to

(9) $$\chi \left(t\right)=1-exp\left[-{\left(\frac{t}{\tau_{0}}\right)}^{\sigma }\right]$$

where

(10) $$\tau_{exp0}={\left(\frac{\sigma }{B}\right)}^{1/\sigma }$$

where $\tau_{exp0}$ and σ are adjustable parameters.

**Figure 4:**
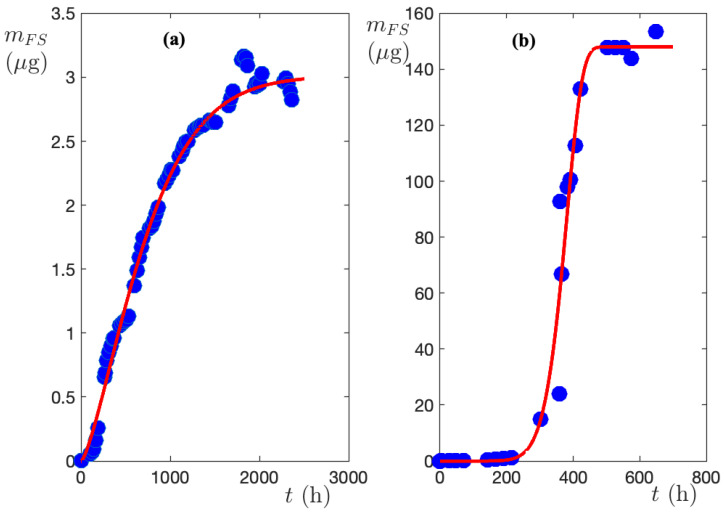
The mass of sodium fluorescein measured in the release bath as a function of time. (**a**) Initial FS = 0.03763 gdm^−3^; (**b**) initial FS = 37.63 gdm^−3^.

Figure 4a displays the $m_{FS}\left(t\right)$ function related to the release from the cylinder initially loaded with FS solution 0.03763 gdm^−3^, while Figure 4b shows the results obtained from the cylinder loaded with FS solution 37.63 gdm^−3^, that is to say, 1000 times larger. It is immediately seen that although the initial concentrations are so different, the model fits to each set of experimental results. The Levenberg–Marquardt procedure [42], used to determine the model parameters, fits the experimental results with a correlation coefficient $R^{2}=0.99$ and root mean square error RMSE=0.03. The calculated parameters are collected in Table 1.

**Figure 5:**
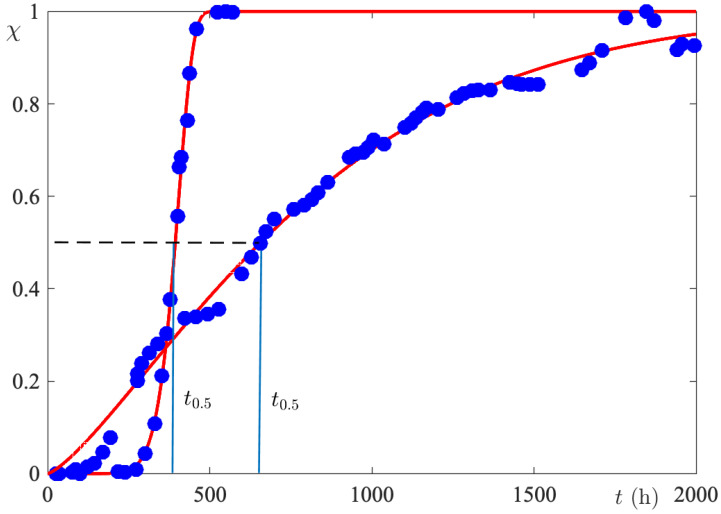
A comparison of kinetic curves obtained from initial FS concentrations which differ by three orders of magnitude. The kinetics are quantitatively compared through the half-release time.

By comparing Figure 4a,b, it becomes evident that the initial concentration strongly influences the rate of release. In order to make the comparison quantitative, normalized release curves (χ(t)) were calculated using the $m_{F}^{\infty }$ parameter given in Table 1, for both investigated systems. From the dimensionless release curves, the half-release time, $t_{0.5}$, i.e., the time required to release half of the maximum amount, was calculated. The procedure displayed in Figure 5 gives $t_{0.5}\left(0.03763\right)=656$ h and $t_{0.5}\left(37.63\right)=395$ h. So, by increasing the initial concentration by three orders of magnitude, $t_{0.5}$ is reduced by 5/6. Since the membrane and release bath volumes are the same in the two sets of experiments, 5/6 is also the ratio of the membrane lifetimes of the FS. The very low variability of $\tau_{exp}$ with initial dye concentration is a manifestation of the strong nonlinearity of the release process.

Accordingly, if one calculates the yield of release from a cylinder loaded with FS 0.03763 gdm^−3^, it is found to be 12%, while for release from a cylinder containing FS 37.63 gdm^−3^, the yield drops dramatically to 0.8%.

In order to understand these results, one has to take into account that dyes in aqueous solutions aggregate, forming molecular species ranging in size from dimers to high-ordered aggregated molecules. Such aggregates not only fail to diffuse, but also prevent individual molecules from crossing the membrane. It is noteworthy that FS is a water-soluble dye that in the concentration range 0.03763–37.63 gdm^−3^ forms H-aggregates, i.e., structures where the dye molecules stack sandwich-like [43]. These structures, both in shape and size, are very different from micelles and can be detected are spectrophotometrically by a blue shift in their absorbance [44]. We point out that the extrusion of PLA at 210 °C can result in a change in mechanical properties due to the thermal degradation of the polymer, making it more brittle. Optical properties also may change due to the loss of transparency after extrusion [45]. Finally, extrusion conditions affect the rate of crystallization by varying the degree of polymer amorphousness. These characteristics can change the permeability of the initial polymer toward drugs. Here, we analyze only the behavior of the membrane at fixed extrusion operating conditions.

## 4. Conclusions

The parameters for FS dissolution and diffusion in the examined bioprinted cylindrical membranes appear to be regulated by PLA swelling. The polymers are initially too hydrophobic to allow significant water penetration into the membranes. Therefore, FS is not sufficiently mobile to be released. However, the polyesters are cleaved by the tiny amounts of water that penetrate the cylindrical membranes. The PLA becomes increasingly hydrophilic with time because the polymer end groups are hydrophilic (–COOH ended). Thus, substantial amounts of water enter after a specific threshold range is crossed. This fundamentally modifies the FS environment. Drug release is made possible by FS dissolution and diffusion through the greatly inflated cylindrical PLA membranes. Future research on the possible effects of the composition, geometry, and size of the membranes on PLA swelling for drug release would be intriguing. As a whole, kinetic measurements show that at low concentrations of dye, where its degree of association is very low, the yield of the release process is 12%. At high concentrations of dye, where its ability to form oligomers is very high, the release yield is 0.8%. This suggests that the release process is not only governed by physical forces but also by the chemical structure of the releasing molecules. It is difficult to formulate a model that takes into account both physical and chemical effects without thereby introducing mathematical adjustable parameters. 

## Figures and Tables

**Table 1:** The parameters of the kinetic model obtained by the Levenberg–Marquardt procedure.

	Dye Initial Concentration 0.03763 gdm^−3^	Dye Initial Concentration 37.63 gdm^−3^
$m_{FS}^{\infty }$	3.0 ± 0.2	147 ± 6
$\tau_{exp0}$	808 ± 4	385 ± 6
σ	1.37 ± 0.09	9 ± 3

## Data Availability

Data will be made available on request.

## Abbreviations

The following abbreviations are used in this manuscript:

FS	fluorescein sodium salt
PLA	polylactic acid
UPW	ultra pure water
RMSE	root mean square error
LOD	limit of determination
LOQ	limit of quantification
